# Roles of exosomal circRNAs in tumour immunity and cancer progression

**DOI:** 10.1038/s41419-022-04949-9

**Published:** 2022-06-09

**Authors:** Baojing Tuo, Zhuang Chen, Qin Dang, Chen Chen, Hao Zhang, Shengyun Hu, Zhenqiang Sun

**Affiliations:** 1 Department of Colorectal Surgery, The First Affiliated Hospital of Zhengzhou University, Zhengzhou, 450052 Henan China; 2 Academy of Medical Sciences, Zhengzhou University, Zhengzhou, 450052 Henan China; 3 School of Life Sciences, Zhengzhou University, Zhengzhou, 450001 Henan China

**Keywords:** Tumour immunology, Cancer microenvironment, Tumour biomarkers, Oncogenes

## Abstract

Tumour immunity plays an important role in the development of cancer. Tumour immunotherapy is an important component of antitumour therapy. Exosomes, a type of extracellular vesicle, act as mediators of intercellular communication and molecular transfer and play an essential role in tumour immunity. Circular RNAs (circRNAs) are a new type of noncoding RNA that are enriched within exosomes. In this review, we describe the effects of exosomal circRNAs on various immune cells and the mechanisms of these effects, including macrophages, neutrophils, T cells, and Natural killer (NK) cells. Next, we elaborate on the latest progress of exosome extraction. In addition, the function of exosomal circRNAs as a potential prognostic and drug sensitivity marker is described. We present the great promise of exosomal circRNAs in regulating tumour immunity, predicting patient outcomes, and evaluating drug efficacy.

## Facts

- Tumour immunity is an important component of the tumour microenvironment and plays an essential role in the development of tumours.
- Exosomes play an important function in intercellular communication.
- Tumour immune escape reduces the efficacy of immunotherapy.
- As a novel marker, exosomal circRNAs have important potential value in predicting tumour progression.

## Open Questions

- In the tumour microenvironment, do various types of immune cells secrete exosomal circRNA to influence other immune cells? Is this effect synergistic or antagonistic?
- Whether immune cells have corresponding mechanisms to fight against the immune escape of tumour cells?
- Is it possible to improve the technology so that high purity exosomal circRNAs can be obtained directly from blood?

## Introduction

Cancer is still an insurmountable problem for humankind [1]. In recent years, tumour immunity has been proven to play an essential role in the development of cancer [2]. Congenital and adaptive immune cells in tumour immunity have made great contributions to inhibiting tumour growth [3, 4], and tumour immunotherapy is receiving increasing attention [5–8]. Recent results, such as immune checkpoint inhibitors, have led to significant advances in tumour immunotherapy [9]. However, due to the complexity of tumour immunity, there are still a series of problems in the specific clinical application of these drugs [10–12].

Exosomes are lipid-binding vesicles secreted by cells into the extracellular space [13]. Exosomes contain a variety of components, such as DNAs, RNAs, lipids, metabolites, cell solutes and proteins, on the cell surface [14]. As the study of exosomes progressed, the functions of exosomes were gradually uncovered by researchers [15]. Exosomes can not only regulate the normal physiological function of the body [16, 17] but also have an important impact on tumour-related biological functions [18]. Exosomes with DNAs, RNAs, proteins and other molecules play an important role in intercellular communication and the exchange of substances [19]. They regulate the functions of the immune system, the nervous system, the cardiovascular system and other important tissues and organs in a normal body [20]. In addition, this intercellular communication plays an important role in tumour development as it can reshape the tumour microenvironment, induce tumour angiogenesis and promote tumour invasion, metastasis and drug resistance [21]. CircRNAs are noncoding RNAs generated by back-splicing that form a closed ring structure by connecting the 3ʹ and 5ʹ ends [22]. They are more stable than linear RNAs due to their special circular covalent bond structure [23]. Recent studies have found that circRNAs are abundant and stable in exosomes [24]. CircRNAs can be transferred from progenitor cells to receptor cells through exosomes and act in receptor cells, affecting tumour progression [25]. For example, exosomal circRNAs can be transferred from tumour cells to immune cells [26], from fibroblasts to immune cells [27], and from macrophages to tumour cells [28, 29]. It has been reported that exosomal circRNAs play an important role in tumour immunoregulation [30]. Interestingly, circRNA has attracted much attention in the early diagnosis of cancer due to its stability, high conservatism, and spatial time-sequence specificity.

In this review, we summarize the role of exosomal circRNAs in tumour immunity and introduce the effects of exosomal circRNAs on various types of immune cells and the mechanism of their function (Table 1). We also highlight their great potential for clinical applications, in particular their ability to predict the efficacy of immune checkpoint inhibitors.

**Table 1:** The role of exosomal circRNAs in tumour immunity.

Cancer types	Source cell	Exosomal circRNA	Receptor cells	Molecular axis	Functions	Result
Oesophageal squamous cell carcinoma	Cancer cell	Circ0048117	Macrophages	circ0048117/miR-140/TLR4	M1-M2	Immune escape
Non-small cell lung cancer		CircFARSA		circFARSA/PTEN/PI3K/AKT		
Hepatocellular carcinoma		Circ0074854				
Glioma		CircNEIL3		CircNEIL3/IGF2BP3/YAP1		
Colorectal cancer		CircPACRGL	Neutrophil	circPACRGL/miR-142-3p/miR-506-3p-TGF-β1	N1-N2	
Hepatocellular carcinoma		CircUHRFI	NK cell	circUHRF1/miR-449c-5p/TIM-3	NK Cell Exhaustion	
Ovarian cancer		Circ0001068	T cell	circ0001068/miR-28-5P/PD1	T cell apoptosis, disintegration, inhibition of proliferation	
Non-small cell lung cancer		CircUSP7		circUSP7/miR-934/SHP2		
Lung adenocarcinoma		CircRNA-002178		circRNA-002178/miR-28-5P/PD1		
			Cancer cell	circRNA-002178/miR-34/PDL1		
Colorectal cancer	Cancer-associated fibroblast	CircEIF3K		circEIF3K/miR-214/PDL1		

### Progress in the isolation of exosomes

With further studies of exosomes, researchers have found that exosomes play essential roles in physiological and pathological functions. Our in-depth study of exosomes has in turn accelerated the development of exosome isolation and extraction technology [31]. Exosomes are typically extracted from blood or cell media. At present, the most commonly used extraction method is ultracentrifugation, also known as the “gold standard” for exosome separation [32]. This method used low-speed centrifugation to remove cell and apoptotic fragments and high-speed centrifugation to extract exosomes from cell metabolites and other substances. Although this method is widely used, it also has some disadvantages, such as a long time requirement, the need for expensive equipment and the low purity of exosome extraction [33]. In addition to centrifugation, many other methods have been developed to extract exosomes, including size exclusion chromatography and ultrafiltration. Researchers found that using a combination of size exclusion chromatography and ultrafiltration produced 58 times more exosomes than ultracentrifugation [34].

Interestingly, in addition to these traditional technologies, researchers have invented new exosome separation technology, called microfluidics [33]. Microfluidics has many advantages over traditional separation techniques, such as low cost and high sensitivity. The technique is designed to capture, filter and isolate exosomes based on their physical and chemical properties [35]. For example, researchers developed a chip that can capture circulating exosomes from serum after modification with anti-CD63 antibodies [36]. The detection of exosomes is based on the extraction of high-purity exosomes. Traditional circRNA detection techniques can be directly applied to exosomal circRNAs, including Northern blot, high-throughput sequencing (HTS), microarray and PCR [31]. Researchers have developed a variety of novel detection methods for exosomal miRNAs [37–39]. The detection technology of exosomal circRNAs remains to be further explored.

### Tumour immunity

Tumour immunity is a complex process that requires the involvement of many cells and molecules [40]. To effectively kill tumour cells, the body’s immune system initiates a series of events, which include filtering dendritic cells to capture and process antigens produced by tumours, activating T cells, T cells specifically identifying and binding cancer cells, and ultimately killing cancer cells [41]. Other immune cells, such as macrophages, also play an important role in fighting tumours [42]. Based on the functions of these immune cells, a variety of therapies have been invented [5]. For example, after coming to a full understanding of the role of dendritic cells in tumours, researchers have developed tumour vaccinations to mobilize antitumour immunity therapy [43]. However, tumour cells can evade the identification and elimination of the immune system and thus continue to survive in the host [44]. In the tumour microenvironment, tumour cells have evolved a variety of ways to evade the immune system, including inducing regulatory immune cells, reducing tumour antigen expression, releasing immunosuppressive factors and promoting immune tolerance and immune bias [45].

The tumour microenvironment is a complex mixture of malignant and nonmalignant cells, including tumour cells, immune cells, and fibroblasts [46, 47]. Most immune cells are malleable, so when they are shaped by cancer cells in the microenvironment, they are affected and differentiate into different phenotypes, such as M1 and M2 macrophages and N1 and N2 neutrophils [48–50]. These different phenotypes have different functions and may even exhibit opposite effects [51]. In addition, immune checkpoints play an important role in tumour immunotherapy [52]. Their overexpression can deplete the function of immune cells. Immune checkpoints include Cytotoxic T-lymphocyte-associated protein 4 (CTLA4), Programmed cell death protein 1(PD1)/Programmed cell death 1 ligand 1 (PDL1), and T-cell immunoglobulin and mucin domain 3 (TIM-3) [53]. Researchers have developed immune checkpoint inhibitor therapy based on the discovery of immune checkpoints, with amazing clinical results [54]. However, due to the complexity of the mechanism involved, there are still many limitations and shortcomings in the actual use of this process.

### Exosomal circRNAs

Exosomes are members of the extracellular vesicles (EVs) family and an important part of the tumour microenvironment [55]. They are secreted by all cell types and can be detected in body fluids such as plasma, urine, semen, and amniotic fluids [56]. The components transported by exosomes vary according to the source cells [57]. By transporting these substances, exosomes not only mediate intercellular communication [58] but also affect the physiological and pathological functions of receptor cells [59]. In recent years, exosomes in the tumour microenvironment have gradually come to the attention of researchers because they can activate immune responses [60]. They can kill tumour cells by submitting antigens from tumour sources to dendritic cells (DCs) to activate T cells, NK cells or macrophages [61]. For example, exosomes of tumour cells that overexpress Ras-related protein Rab-27 (Rab27) can promote the proliferation of CD4+ T cells, resulting in more effective antitumour immunity [62]. Exosomes rich in miRNA-124 can enhance the antitumour immune response in a colon cancer mouse model [63].

With the discovery of circRNAs in exosomes, the role of exosomal circRNAs in tumours has been gradually revealed. At the same time, it has been found that exosomal circRNAs have an important effect on immune cells in the tumour microenvironment. In 1976, the first circRNA was found in RNA viruses [64]. For decades, it was thought to be the result of a splicing error. For nearly a decade, with the development of RNA-sq technology, more than 30,000 circRNAs have been discovered, and their abundance and diversity are constantly refreshing people’s perceptions. At the same time, the biological occurrence and function of circRNAs have been gradually discovered [65]. They have been found to act as miRNA sponges, interact with proteins, be translated into proteins, and bind RNA polymerase II as transcription factors [66]. Building on these findings, some of the functions of circRNAs in some diseases, especially cancer, are also gradually being revealed [67–71]. For example, studies have found that overexpression of exosomal circSHKBP1 can promote the proliferation, migration, invasion, and angiogenesis of gastric cancer (GC) [72]. Similarly, exosomal circRNA-100338 enhances the transfer capacity of hepatocellular carcinoma (HCC) cells [73]. Moreover, exosomal circRNAs are strongly associated with the growth and proliferation of lung, pancreatic, thyroid, and colorectal cancers [74–77].

### Sources of exosomal circRNAs

In the tumour microenvironment, most cells can produce exosomal circRNAs, such as tumour cells, immune cells, and fibroblasts. Exosomal circRNAs secreted by these cells are different and play important roles in the development of tumour [78–80]. CircRNAs are abundant and stable in exosomes. They are thought to be able to be transported by progeny cells through exosomes to mediate effects on receptor cells [81]. Studies have demonstrated that exosomal circRNAs of tumour origin play an important role in the development of tumours [72, 82, 83]. For example, compared to normal tissues, circNRIP1 is highly expressed in gastric cancer tissue. The study has confirmed a negative correlation between circNRIP1 and miR-149-5p expression levels. Previous reports have shown that miR-149-5p can affect tumour proliferation and invasion by targeting the AKT1/mTOR pathway or in other ways [84, 85]. In the tumour microenvironment, tumour-related macrophages (TAMs) play important roles in tumour immunity and escape. Exosomal circRNAs produced by macrophages can also affect tumour progression [28, 86]. For example, hsa_circ_0004658 is significantly enriched in exosomes produced by macrophages overexpressing RBPJ (recombination signal binding protein-Jκ) and acts as ceRNA for miR-499b-5p, affecting the JAM3 pathway and ultimately inhibiting tumour progression [29]. The matrix of the tumour microenvironment is mainly composed of fibroblasts and other interstate cells, the most important and largest number of which are fibroblasts. In addition, fibroblasts are recognized to have a huge impact on the biological function of cancer [87]. Studies have shown that exosomal circEIF3K from tumour-related fibroblasts can promote tumour immune escape and accelerate tumour progression. This is of great significance to the study of the effect of tumour interstate cells on tumour development. The above shows that exosomal circRNAs of different cellular origin in the tumour microenvironment are different and produce effects in different ways.

Interestingly, in addition to the cells mentioned above, there are also cells that are capable of secreting exosomal circRNAs, and although they are not currently known to have an effect on tumours, they still have some important physiological roles. For example, the osteoblast-derived exosomal circ_0008542 is able to bind competitively to miRNA-185-5p, thereby promoting osteoclast-induced bone resorption [88]. In addition, the stem cell-derived exosome circHIPK3 can promote the repair of ischaemic muscle damage and exosomal circRNA can be selectively released in platelets [89, 90].

### Effects of exosomal circRNAs on tumour-associated macrophages (TAMs)

Macrophages are widely present in all tissues and exhibit great functional diversity [91]. Most solid tumours are inundated with large amounts of macrophages, known as tumour-associated macrophages (TAMs) [92]. They play essential roles in fighting pathogens, tissue damage, and repair [93, 94]. Macrophages can be induced to activate and differentiate into a wide variety of phenotypes under the influence of the surrounding environment. M1- and M2-polarized macrophages can be understood as extremes of activation state continuums in this adaptive response field [95]. There is growing evidence that exosomal circRNAs are involved in the regulation of macrophages in the tumour microenvironment. For example, in oesophageal squamous cell carcinoma (ESCC), exosomal hsa-circ-0048117 is highly expressed in exosomes secreted by oxygen-deprived tumour cells. After incubation with exosomes, researchers found significant increases in the amounts of Arg1, IL-10, and TGF-β, which are secreted mainly by M2 macrophages. In addition, they found that overexpression of hsa-circ-0048117 can increase the expression of toll-like receptor 4 (TLR4) [96]. TLR4 has been proven to promote the proliferation of M2 macrophages [97]. Existing studies have shown that M2 macrophages tend to act as immunosuppressive agents, removing debris and promoting angiogenesis, tissue repair, and tumour progression [98].

**Fig. 1:**
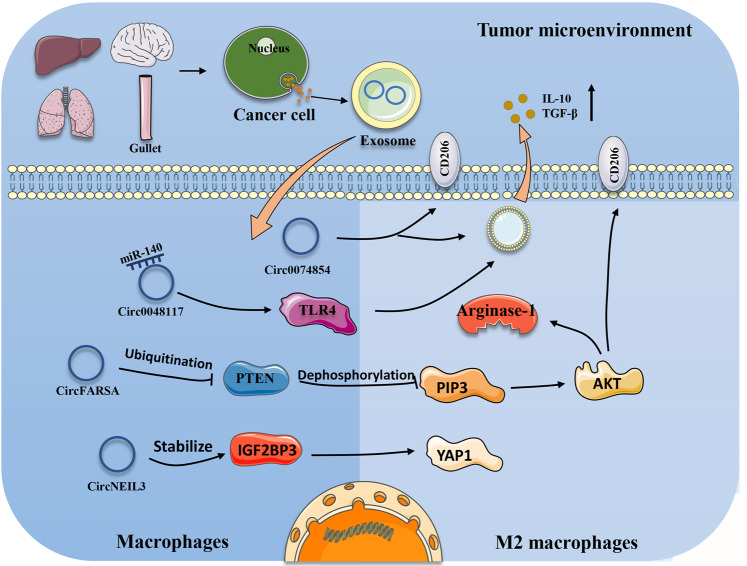
Diagram of the effects of exosomal circRNAs on macrophages. CircRNAs are transported into macrophages through exosomes. They sponge certain miRNAs to improve the expression of the genes involved. They also affect signalling pathways to function. The end result is to regulate macrophage M2 polarization.

In addition, in non-small-cell lung cancer (NSCLC), researchers found that macrophages cultured with exosomes secreted by tumour cells overexpressed circFARSA. CircFARSA increases the expression of Arginase-1 and CD206 after it enters macrophages, suggesting that circFARSA can induce macrophages to M2 polarization [26]. Furthermore, exosomes from HepG2 cells can enter macrophages, causing macrophages to exhibit a CD86^low^/CD206^high^ phenotype, suggesting that exosomes of tumour cell origin can promote macrophage polarization to the M2 phenotype. Interestingly, in HepG2 cells treated with exosomes with low hsa_circ_00074854 expression, the levels of Zinc-finger E-box binding homeobox 1 (ZEB-1) and vimentin were significantly higher and E-cadherin levels were lower. Therefore, exosomal hsa_circ_00074854 can affect the progression of HCC by affecting macrophage phenotypes [99]. Glioma cells are able to produce the exosome circNEIL3 and transport it into macrophages. It binds to IGF2BP3 and prevents its ubiquitination, resulting in increased expression of IGF2BP3. Previous studies have shown that YAP1 expression can contribute to the conversion of macrophages to the M2 phenotype [100]. IGF2BP3 can increase the expression of YAP1. In summary, exosomal circRNAs promote the M2 polarization of macrophages, thereby affecting the growth and development of tumour cells and metastasis (Fig. 1).

### Effects of exosomal circRNAs on immune checkpoints and T cells in cancer

Cancer immune escape is a key factor affecting cancer treatment [101]. It is generally believed that the occurrence of immune escape of tumour cells goes through three stages: elimination, balance, and escape [102]. Tumour cells can achieve immune escape by improving the expression levels of immune checkpoint proteins, such as PD-1 and PDL1 [103]. Recent studies have shown that exosomal circRNAs can enhance the expression of immune checkpoint proteins in tumour cells or immune cells, thereby affecting the ability of immune cells to fight the tumour. For example, studies found that the expression of PD1 was significantly increased in T cells incubated with exosomes containing circ-0001068 [104]. PD1 is expressed on activated T cells, and its corresponding ligand, PDL1, can be expressed on a variety of cells [105]. When PD1 is combined with PDL1, a series of reactions are produced to inhibit the activation, proliferation, survival, and cytotoxic secretion of T cells, leading to immune escape [106].

**Fig. 2:**
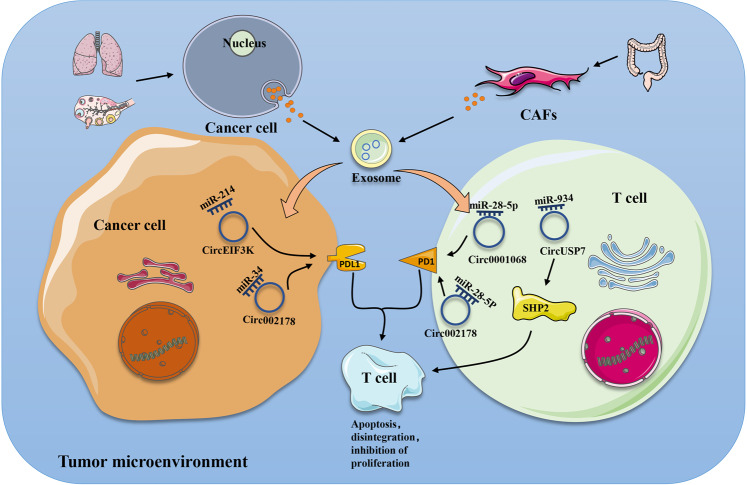
Effects of exosomal circRNAs on immune checkpoints and T cells. CircRNAs sponge specific miRNAs as they enter cancer cells or T cells, increasing PDL1/PD1 expression. After PD1 was combined with PDL1, the activity and proliferation of T cells decreased significantly.

Furthermore, one study showed a significant increase in circRNA-002178 in lung adenocarcinoma (LUAD) tissue compared to adjacent nontumour tissues. They found that knocking down circRNA002178 significantly reduced the activity of luciferase carrying PDL1 3’UTR sequences, and the expression of PDL1 decreased significantly in 95D cells transfected with circRNA-002178 siRNA. Interestingly, circRNA002178 can also enter CD8+ T cells to increase the expression of PD1. In the latest study, the number of CD8+ T cells in patients with non-small-cell lung cancer was significantly related to the expression of circUSP7 in plasma exosomes. They then demonstrated that exosomal circUSP7 affected the expression level of SHP2 in CD8+ T cells, leading to CD8+ T cell dysfunction [107]. In addition, exosomal circEIF3K can affect the expression of PDL1 in CRC. After hypoxia treatment of carcinoma-associated fibroblasts (CAFs), they found an increase in the expression of exosomes containing circEIF3K. These exosomes enter tumour cells and promote the expression of PDL1 [27]. These results show that exosomal circRNAs play an irreplaceable role in the expression of immune checkpoints and the function of T cells (Fig. 2).

### Effect of exosomal circRNAs on natural killer cells in cancer

**Fig. 3:**
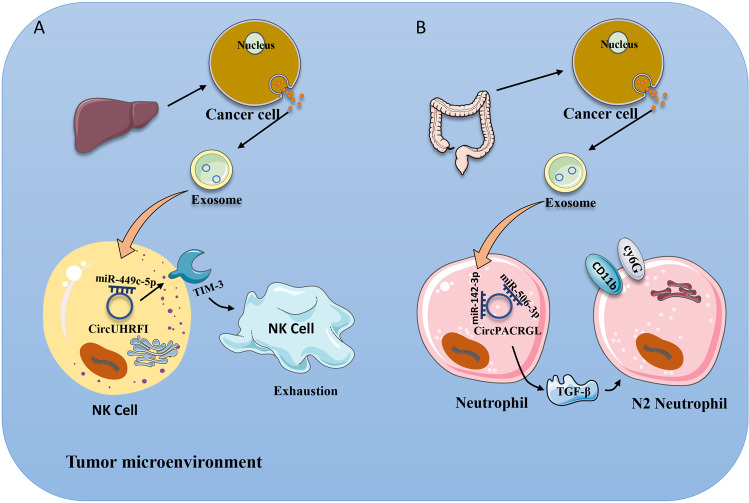
Effects of exosomal circRNAs on NK cells and neutrophils. **A** Exosomal circPACRGL derived from cancer cells sponges miR-449c-5p to increase the expression of TIM-3. This results in the exhaustion of NK cells. **B** Exosomal circPACRGL sponges miR-142-3P or miR-506-3P and increases the expression of TGF-β. This leads to an increase in the neutrophil N2 phenotype.

NK cells are of great concern because of their powerful ability to kill tumour cells and their proinflammatory role [108]. They have a wealth of functions and are widely present in various organs of the human body [109]. NK cells can play a cytotoxic role, identify and kill infected and malignant cells, and secrete cytokines to regulate other immune cells [110]. In the process of fighting tumours, NK cells identify tumour cells in two ways: one is to identify tumour cells that do not express human leukocyte antigen class I (HLA-1) molecules [111], and the other is to activate the toxicity of NK cells by increasing the levels of damage-related proteins [112]. Recently, researchers have made discoveries about the mechanisms that affect NK cells in their fight against tumours. The results showed that circUHRF1 was highly expressed in HCC, while patients with high expression of circUHRF1 had larger tumour volumes and smaller numbers of NK cells in the blood. In addition, they proved that circUHRF1 increased the expression of Tim-3 [113]. TIM-3, an emerging tumour immune checkpoint, is expressed in NK cells as well as other immune cells [114]. Highly expressed TIM-3 is associated with poor prognosis [115]. Blocking Tim-3 and PD1 together was found to achieve better results [116]. In summary, exosomal circUHRF1 promotes the expression of TIM-3 in NK cells, resulting in the depletion of NK cell function and the promotion of tumour progression (Fig. 3A).

### Effect of exosomal circRNAs on tumour-associated neutrophils (TANs)

Neutrophils are an important part of the body’s innate and humoral immunity [117]. Similar to TAMs, tumour-associated neutrophils (TANs) are also divided into two types, N1 and N2 [118]. N1 has cytotoxicity and antitumour effects, and the N2 type promotes tumour progression [119]. In recent years, the exploration of the impacts of exosomes on neutrophils has continued. Exosomes have been shown to promote the polarization of neutrophils to N2 or the migration of neutrophils, thus affecting the progression of tumours [120–122]. For example, extracellular vesicles derived from gastric cancer cells can increase the expression of PDL1 in neutrophils, which is achieved by activating signal transducer and activator of transcription 3 (STAT3).

Interestingly, one study reported that transfecting circPACRGL into CRC cells resulted in the proliferation and migration of tumour cells. At the same time, researchers noted that exosomal circPACRGL affects the expression of transforming growth factor‑β (TGF-β). There are reports that TGF-β plays an important role in polarizing neutrophils into N2 phenotypes [123]. The percentage of N2 neutrophils decreased significantly in the circPACRGL knockdown cell group, while overexpression of TGF-β1 reversed this process [124]. Therefore, exosomal circPACRGL promotes the polarization of neutrophil N2, which leads to the immune escape of tumours [125] (Fig. 3B).

### Mechanism of exosomal circRNAs in tumour immunity

The above studies have proven that exosomal circRNAs play a significant role in tumour immunity. The ways in which exosomal circRNAs function in tumour-associated immune cells are different. For example, exosomal circFARSA promotes PTEN ubiquitination and degradation. Researchers have found that circFARSA transfection into macrophages affects phosphatase and tensin homolog deleted on chromosome ten (PTEN) protein levels in a dose-dependent manner. Similarly, the presence of circFARSA reduces the half-life of PTEN. Previous studies have demonstrated that PTEN inhibits the PI3K/AKT pathway, which is achieved by dephosphorylating PIP3 and inactivating AKT [126]. The PI3K/AKT pathway plays an important role in the M2 polarization of macrophages [127]. Overall, circFARSA affects macrophage M2 polarization by affecting the expression of PTEN [26].

In 2011, Salmena et al. formally proposed the “ceRNA” hypothesis. In this hypothesis, noncoding RNAs such as lncRNAs are thought to be endogenous competitive RNAs that compete with real coding RNAs to bind miRNAs. This will inhibit the silent effect of miRNAs on encoded RNAs [128]. Although this hypothesis is controversial, it is also accepted by most researchers [129]. Subsequently, it was found that circRNAs also apply to the ceRNA hypothesis. They act as sponges to regulate the proliferation, apoptosis, invasion, and migration of tumour cells [129, 130]. For example, hsa-circRNA-104348, as a ceRNA, combines with miR-187-3p to hinder its function and affect the progression of hepatocellular carcinoma [131]. In addition, circRNAs can also be transported through exosomes to receptor cells to play a role. In ESCC, exosomal circ0048117 can act as a sponge of miR-140, affecting its function. The target gene of miR-140 is TLR4, which has been shown to promote macrophage M2 polarization [96, 132]. Researchers found that circRNA-002178 also acts as a ceRNA of miR-34a. In highly expressed LUAD tissues, the expression of miR-34a is significantly lower [133]. In addition, in colorectal, liver, and ovarian cancers, exosomal circRNAs act as ceRNAs to play a role, thereby affecting the proliferation and development of tumour cells [27, 107, 113, 125].

### Emerging clinical benefits

Cancer is a major problem for human health [134]. However, early detection methods for cancer lack sensitivity and specificity [135]. CircRNAs have been shown to act as emerging tumour markers [136]. Recently, a large number of circRNAs have been found in exosomes. The functions of some exosomal circRNAs in tumour immunity have been confirmed, and these circRNAs are potential prognostic markers and therapeutic targets. For example, recent research mentioned that exosomal circFARSA is highly expressed in the plasma of patients. This expression may be used to predict the prognosis of patients with NSCLC [26]. In addition, by comparing the sera of ESCC patients with healthy volunteers, it was found that hsa-circ-0048117 was highly expressed in the serum and that exosomal hsa-circ-0048117 was associated with TNM grading of tumours [96].

In the past 10 years, the discovery of immune checkpoints, especially for PD1/PDL1 pathway blockade, has been the largest discovery of immunotherapy for cancer, which has taken immunotherapy to a new level. Recent studies have shown that exosomal circRNAs may be able to predict the efficacy of immune checkpoint inhibitors. They can affect the expression of PD1/PDL1 or otherwise affect the efficacy of immune checkpoint inhibitors. For example, exosomal circRNA-002178 can enter CD8+ T cells to promote the expression of PD1 [133]. In addition, the exosomal circEIF3K secreted by cancer-related fibroblasts has also been shown to be associated with the expression of PDL1 [27]. In ovarian cancer, after incubation with A2780 cells that secrete exosomal circ-0001068, the expression of PD1 in T cells increased significantly [104]. Adjusting the expression of PD1/PDL1 is beneficial to the efficacy of antitumour drugs [137]. Interestingly, the researchers treated HunSG mice that expressed different amounts of exosomal circUSP7 with PD1 antibodies. The results showed that xenograft mice with high expression of exosomal circUSP7 demonstrated significant resistance [107]. Similarly, excessive expression of circUHRF1 may lead to liver cancer resistance to PD1 treatment, while targeting circHRF1 may inhibit this process [113]. In summary, exosomal circRNAs can be used as markers of tumour prognosis but can also guide immune checkpoint inhibitors. They can also act as drug targets to enhance the sensitivity of immunotherapy.

## Conclusions and perspectives

Tumour immunity has always been an important direction for researchers to study in the treatment of tumours. The related function of exosomal circRNAs in tumour immunity has also been gradually revealed. As an important medium of intercellular communication, exosomes are also natural carriers of transport signal molecules, which play an important role in tumour immunity. This paper summarizes the roles of exosomal circRNAs in various types of immune cells in the tumour microenvironment and the detailed mechanisms by which they function. Based on the properties of the exosomal circRNAs, we have discovered their advantages as markers of tumour diagnosis and prognosis, as well as their ability to judge the efficacy of targeted drugs.

In recent years, the advent of engineered exosome technology has allowed exosomes to carry a range of contents, including peptides and miRNAs [138]. On this premise, based on the function of the exosomal circRNAs currently known, we propose whether a particular circRNA can be placed in large quantities in exosomes. These circRNAs could be carried into target cells to affect the expression of PD1/PDL1 to increase the sensitivity to targeted drugs. At present, although great progress has been made in cancer immunotherapy-related research, there are still several problems. In the current studies, researchers discovered the effects of exosomal circRNAs on macrophages, neutrophils, NK cells, and the ability to deplete T cells by affecting immune checkpoints. However, other immune cells, such as B cells and myeloid-derived suppressor cells (MDSCs), have been proven to play important roles in tumour immunity [139–141]. The effects of exosomal circRNAs on these cells remain unknown. Although exosomal circRNAs show great potential for clinical application, there is still a long way to go before they can be applied to clinical practice due to current technical limitations.

## Data Availability

The datasets employed or/and scrutinized within the present investigation are accessible from the corresponding author on reasonable requests.
